# Transcription factor 7-like 1 dysregulates keratinocyte differentiation through upregulating lipocalin 2

**DOI:** 10.1038/cddiscovery.2016.28

**Published:** 2016-04-25

**Authors:** M Xu, Y Zhang, H Cheng, Y Liu, X Zou, N Zhan, S Xiao, Y Xia

**Affiliations:** 1 Department of Dermatology, The Second Affiliated Hospital, School of Medicine, Xi'an Jiaotong University, Xi’an 710004, China; 2 Intensive Care Unit, China Gezhouba Group Central Hospital, The Third Clinical Medical College of China Three Gorges University, Yichang, China; 3 Department of Medicine, The Second Affiliated Hospital, School of Medicine, Xi'an Jiaotong University, Xi’an, China; 4 Department of Dermatology, Hubei Maternity and Child Health Hospital, Wuhan, China; 5 Department of Pathology, Renmin Hospital of Wuhan University, Wuhan, China

## Abstract

Recent studies strongly suggested that transcription factor 7-like 1 (Tcf7l1, also known as Tcf3) is involved in the differentiation of several types of cells, and demonstrated that Tcf7l1 modulates keratinocytes physiologically through regulating lipocalin 2 (LCN2), a key regulator of cell differentiation. To reveal the potential role of Tcf7l1 in the dysregulation of keratinocyte differentiation, both Tcf7l1 and LCN2 were determined in a variety of skin disorders. The *in vitro* effect of Tcf7l1 on keratinocyte differentiation was studied by culturing SCC-13 cells, and the human foreskin keratinocytes (HFKs) that were transfected with vectors for overexpressing human papillomavirus E6/E7 or Tcf7l1 genes. We found that both Tcf7l1 and LCN2 were highly expressed in those diseases characterized by defective keratinocyte differentiation (especially psoriasis vulgaris, condyloma acuminatum, squamous cell carcinoma, etc). Moreover, compared with control HFKs, SCC-13 cells and E6/E7-harboring HFKs expressed more Tcf7l1 and LCN2. Tcf7l1 siRNA transfection decreased LCN2 but increased involucrin and loricrin in HFKs under calcium stimuli. Conversely, Tcf7l1 overexpression in SCC-13 cells or vector-transfected HFKs induced lower involucrin and loricrin expression and less keratinocyte apoptosis, both of which, however, were partially abrogated by LCN2 siRNA or neutralizing anti-LCN2 antibody. Interestingly, the Tcf7l1 expression in HFKs correlated positively with the MMP-2 level, and the inhibition of MMP-2 decreased the LCN2 level and even attenuated the effect of Tcf7l1 on LCN2 expression. Therefore, Tcf7l1 dysregulates keratinocyte differentiation, possibly through upregulating the LCN2 pathway in an MMP-2 mediated manner. Elucidating the interaction between Tcf7l1 and LCN2 may help understand disordered cell differentiation in some skin diseases.

## Introduction

Transcription factor 7-like 1 (Tcf7l1), also called the high mobility group box transcription factor Tcf3, is a key regulatory protein that institutes transcriptional programs for cell development and proliferation.^1–3^ Tcf7l1 is expressed by a variety of cell types, including neural and follicular stem cells, epidermal cells and cancer cells of multiple origins.^2,4–7^ Through direct binding to multiple gene sites and interaction with proteins, Tcf7l1 regulates Wnt-*β*-catenin and c-Myc signaling, which further functions in the differentiation, proliferation and malignancy of different cells.^8,9^ Recently, Tcf7l1 activation was found in cutaneous keratinocytes, especially those with a poor differentiation status.^4,8,10^ Induction of TCF7L1 in mouse epidermal progenitor cells is sufficient to abrogate terminal differentiation and maintain an undifferentiated cell fate.^4^ Moreover, Tcf7l1 is upregulated in skin wounds, and then accelerates keratinocyte migration and skin wound healing through regulation of lipocalin 2 (LCN2, or neutrophil gelatinase-associated lipocalin).^11^ Many studies have demonstrated that LCN2 signals play an important role in cell differentiation.^12–14^ Therefore, Tcf7l1 signaling is pivotal in the modulation of keratinocyte behaviors especially differentiation.

LCN2 is an autocrine cytokine functioning through interaction with the LCN2 receptor that is expressed in various cells. By multiple mechanisms, LCN2 is involved in cell differentiation. LCN2 inhibits the nuclear factor *κ*B pathway,^14^ activates the Met/FAK cascade,^15^ upregulates mesenchymal markers but downregulates the epithelial marker E-cadherin,^16^ and also gives rise to cells with a more immature phenotype.^17^ Moreover, LCN2 is strongly expressed in cancer cells and correlates negatively with the differentiation grade of tumors.^15,16,18^ LCN2 forms complex with both MMP-2 and MMP-9 in cancer cells,^19–21^ which are also suggested to be functional in regulating cell differentiation.^22,23^ Interestingly, LCN2 expression is promoted in keratinocytes upon HPV infection that confers keratinocytes property of poor differentiation.^24^ LCN2 even fluctuates with the differentiation degree of keratinocytes and preferentially appears in differentiating keratinocytes or some skin diseases with parakeratotic epidermis.^25,26^ All these findings strongly indicated LCN2 as a marker for dysregulated keratinocyte differentiation in skin. However, the upstream regulation of LCN2 signals, especially that involves cell differentiation, remained unclear to date.

The dysregulated differentiation of keratinocytes is commonly seen in several skin disorders including psoriasis, warts and squamous cell carcinoma (SCC). To unveil the mechanisms related to such dysregulation is important in the development of novel therapeutic approaches for the treatment of these diseases. Considering the facts that Tcf7l1 closely modulates the behaviors of keratinocytes and regulates the LCN2 pathway under certain condition,^11^ we presumed that Tcf7l1 also play a role in the dysregulation of keratinocyte differentiation. Therefore, the aim of this study was to investigate the expression Tcf7l1 in skin diseases and the possible mechanism underlying its regulation of keratinocyte differentiation.

## Results

### Both Tcf7l1 and LCN2 are highly expressed in skin diseases featuring dysregulated keratinocyte differentiation

**Figure 1:**
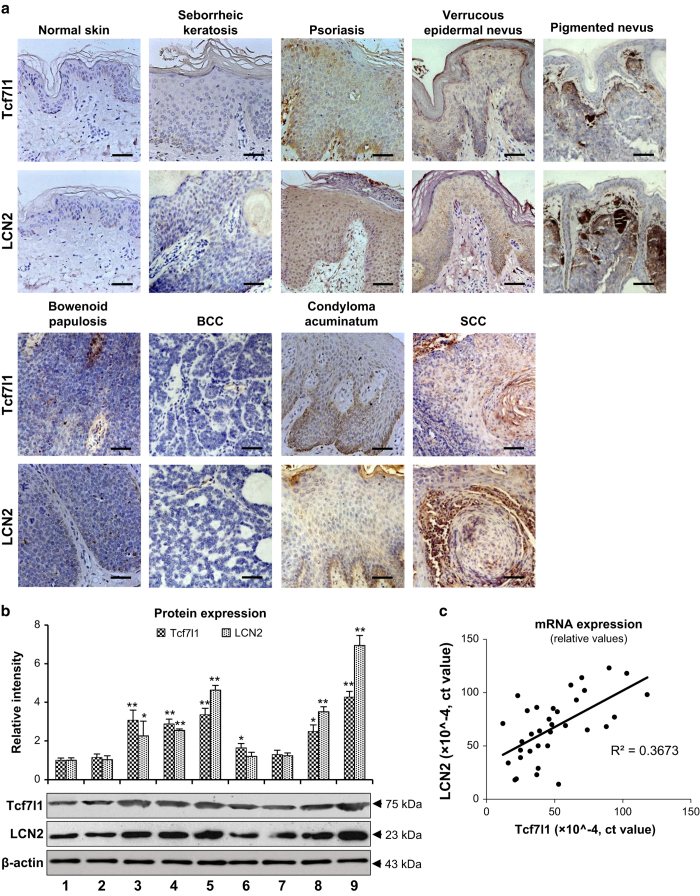
Tcf7l1 and LCN2 expression in skin diseases. (**a**) By immunohistochemistry, Tcf7l1 and LCN2 were stained in normal skin (*n*=4), seborrheic keratosis (*n*=4), psoriasis vulgaris (*n*=5), verrucous epidermal nevus (*n*=3), pigmented nevus (*n*=4), Bowenoid papulosis (*n*=3), BCC (*n*=4), condyloma acuminatum (*n*=4) and SCC (Broder’s grade I, *n*=3). (**b**) By western blot, the Tcf7l1 and LCN2 proteins were detected individually in all tissue lysates, followed by a quantitation of band intensities using ImageJ. Numbers of 1 to 9 indicate the normal skin and eight diseases shown in (**a**) in order. *P<0.05, **P<0.01, compared with normal skins accordingly. Data are expressed as mean±S.E.M. Statistical differences were tested with one-way ANOVA followed by a two-tailed *t*-test. (**c**) The mRNA expression levels were determined individually, followed by the linear correlation between the Tcf7l1 and LCN2 values (*n*=34, *P*<0.05). Linear regression was done for the correlation between the mRNA values of Tcf7l1 and LCN2. Representative images are shown. Scale bar=50 *μ*m.

By immunohistochemistry, both Tcf7l1 and LCN2 were strongly expressed in psoriasis vulgaris, verrucous epidermal nevus, condyloma acuminatum and SCC (Broders’ grade I) while negative or slight expression was seen with normal skins, seborrheic keratosis, Bowenoid papulosis and basal cell carcinoma (BCC) (Figure 1a). In the four diseases that showed stronger Tcf7l1 staining, western blotting revealed higher levels of Tcf7l1, which were usually accompanied by elevated LCN2 expression (Figure 1b). Moreover, we determined tissue mRNA expression of these two proteins in the samples, and found a positive correlation between them (Figure 1c). Pigmented nevus also exhibited both Tcf7l1 and LCN2 overexpression, which was predominant in melanocytes underneath the epidermis but not in keratinocytes (Figures 1a and b).

Tcf7l1 expression was even evaluated in SCC tissues that were classified based on cell differentiation. In both protein and mRNA levels, Tcf7l1 increased in terms of Broders’ classification grade (Supplementary Figure S1).

### Both Tcf7l1 and LCN2 increase in keratinocyte-originated cells with differentiation dysregulation

**Figure 2:**
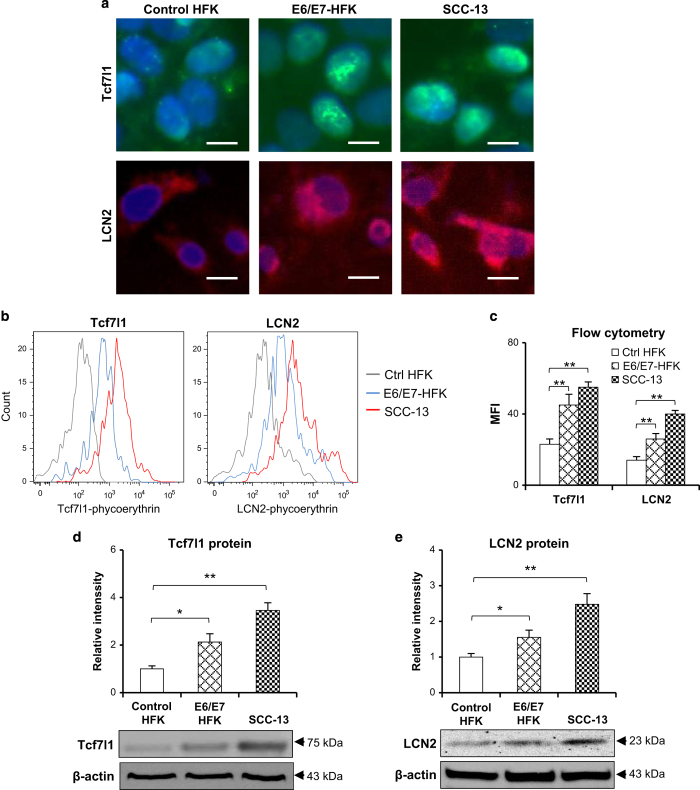
Tcf7l1 and LCN2 expression in cells *in vitro*. Control HFKs (transfected with blank vector), E6/E7 gene-transfected HFKs, and SCC-13 cells were cultured *in vitro*. (**a**) Immunofluorescence showed the strongest Tcf7l1 (green) and LCN2 (red) staining in SCC-13 cells, followed by E6/E7-transfected and control HFKs in order. (**b** and **c**) Flow cytometry confirmed more Tcf7l1 and LCN2 expression in SCC-13 cells and E6/E7-transfected HFKs when compared with control HFKs. MFI, mean fluorescent intensity. (**d** and **e**) Western blot revealed the highest levels of Tcf7l1 (**d**) and LCN2 (**e**) in SCC-13 cells. Data show mean±S.E.M. of triplicate cultures (*n*=3). Statistical differences were tested with one-way ANOVA followed by a two-tailed *t*-test. Representative images are shown. Scale bar=5 *μ*m. *P<0.05; **P<0.01.

**Figure 3:**
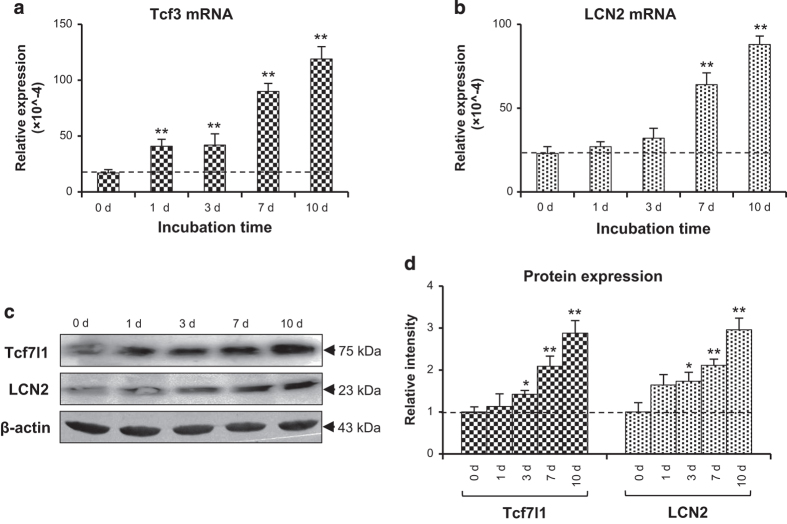
Tcf7l1 and LCN2 expression in keratinocytes undergoing differentiation. HFKs were cultured *in vitro* with the addition of calcium (1.2 mM). (**a** and **b**) The mRNA levels of Tcf7l1 (**a**) and LCN2 (**b**) were determined in HFKs that were harvested at different time points. (**c** and **d**) Western blot was performed with protein extracts of HFKs for Tcf7l1 and LCN2 expression (**c**), followed by quantitation of band intensities with ImageJ (**d**). Data show mean±S.E.M. of triplicate cultures (*n*=3). Statistical differences were tested with one-way ANOVA followed by a two-tailed *t*-test. Representative images are shown. *P<0.05, **P<0.01, compared with the values on day 0 accordingly.

The expression of Tcf7l1 and LCN2 was investigated further in cells that differed in differentiation. Immunofluorescent detection showed that, compared with control (transfected with blank vector) human foreskin keratinocytes (HFKs), the SCC-13 cells or HPV16 E6/E7-transfected HFKs expressed more Tcf7l1 and LCN2 (Figure 2a), which was even quantitatively confirmed by flow cytometry (Figures 2b and c). Proteins were extracted from these cells, displaying elevated levels of Tcf7l1 and LCN2 in the SCC-13 and E6/E7-positive cells (Figures 2d and e). The differences in differentiation between these cells were verified by assessing two specific markers (involucrin and loricrin), showing lower levels in E6/E7-positive cells and SCC-13 cells when compared with control HFKs (Supplementary Figure S2). Moreover, HFKs were induced for differentiation by calcium stimuli, followed by mRNA and protein extraction at different time points. The results showed that Tcf7l1 and LCN2 expression increased with incubation time of calcium (Figure 3).

### Tcf7l1 depletion represses LCN2 expression during differentiation of HFKs

**Figure 4:**
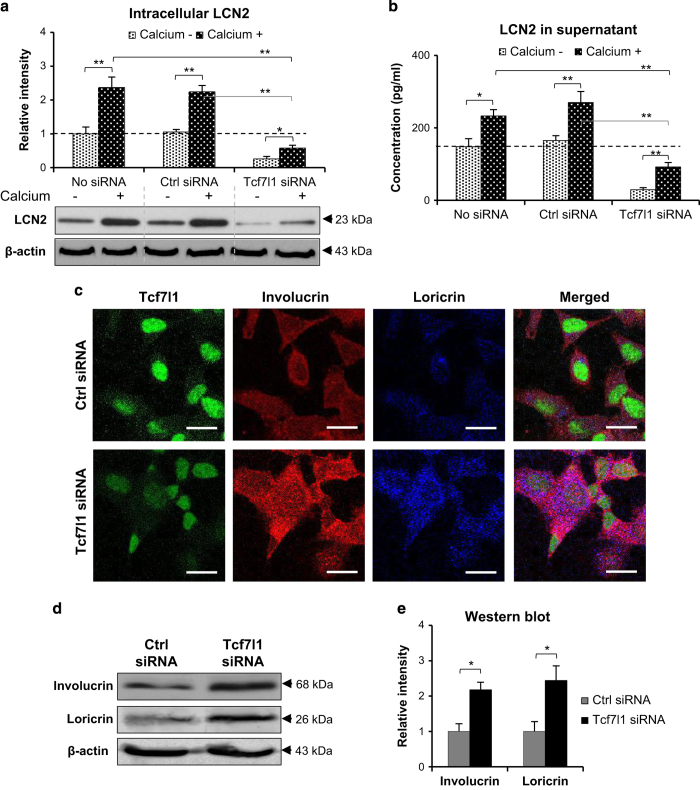
The effect of Tcf7l1 depletion on LCN2 expression in HFKs. HFKs were stimulated with calcium chloride (1.2 mM, 7 days) after siRNA transfection. (**a** and **b**) Intracellular (**a**) and soluble (**b**) LCN2 levels were evaluated by western blot and ELISA, respectively. (**c**) By immunofluorescence, the expression of involucrin and loricrin were detected in HFKs that were transfected with Tcf7l1 or control siRNA. (**d** and **e**) The expression of involucrin and loricrin was also detected in HFKs by western blot. Data show mean±S.E.M. of triplicate cultures (*n*=3). Statistical differences were tested with one-way ANOVA followed by a two-tailed *t*-test. Representative images are shown. Scale bar=10 *μ*m. *P<0.05; **P<0.01.

To demonstrate the direct effect of Tcf7l1 on LCN2 expression and keratinocyte differentiation, HFKs were transfected with Tcf7l1 siRNA and then stimulated with calcium chloride (1.2 mM, 7 days). It was found that Tcf7l1 but not control siRNA reduced Tcf7l1 synthesis substantially (Supplementary Figure S3). The intracellular and soluble (in culture supernatants) LCN2 was determined by western blot and enzyme-linked immunosorbent assay (ELISA), showing remarkable reduction upon Tcf7l1 siRNA transfection (Figures 4a and b). Flow cytometry further confirmed such reduction of intracellular LCN2 (Supplementary Figure S3). Despite of siRNA transfection, calcium stimuli enhanced LCN2 expression in HFKs (Figures 4a and b). Moreover, both immunofluorescence and western blot demonstrated more expression of involucrin and loricrin by the HFKs transfected with Tcf7l1 siRNA (Figures 4c–e). Consistently, such increase in involucrin and loricrin expression was also verified in these HFKs by quantitative real-time polymerase chain reaction (qRT-PCR) (Supplementary Figure S3).

### Tcf7l1 overexpression promotes LCN2 level but inhibits apoptosis of HFKs undergoing differentiation

**Figure 5:**
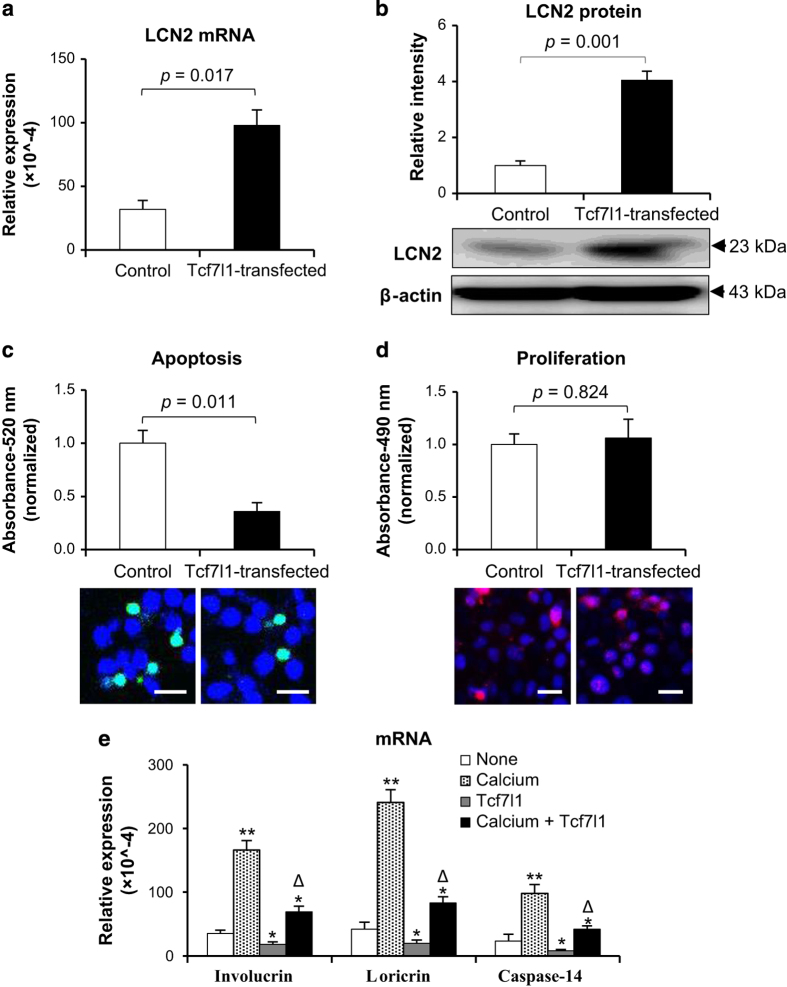
The effect of Tcf7l1 overexpression on HFKs. After the transfection of Tcf7l1-inserted pcDNA vector, HFKs were stimulated with calcium chloride (1.2 mM, 7 days). (**a** and **b**) By qRT-PCR (**a**) and western blot (**b**), HFKs expressed more lipocalin 2 (LCN2) upon Tcf7l1 pcDNA transfection. (**c** and **d**) Both cell apoptosis (**c**) and proliferation (**d**) were determined in HFKs that were either transfected with Tcf7l1 pcDNA or not. Cell apoptosis and proliferation were also visualized by TUNEL and fluorescent Ki-67 staining, respectively. (**e**) qRT-PCR was performed for the mRNA levels of involucrin, loricrin and caspase-14 in HFKs. Data show mean±S.E.M. of triplicate cultures (*n*=3). Statistical differences were tested with one-way ANOVA followed by a two-tailed *t*-test. Scale bar=10 *μ*m. In (**e**), *P<0.05, **P<0.01, compared with nontreated groups accordingly; ^Δ^*P*<0.05, compared with calcium alone-treated HFKs accordingly.

HFKs were transfected with a lentiviral vector to overexpress Tcf7l1 (Supplementary Figure S4), followed by calcium stimulation. By qRT-PCR and western blot, it was found that LCN2 increased in Tcf7l1-overexpressing HFKs when compared with the non-Tcf7l1-transfected HFKs (Figures 5a and b). Moreover, compared with the same control, Tcf7l1-transfected HFKs exhibited a significant decrease in apoptosis while cell proliferation was not affected (Figures 5c and d). Additionally, Tcf7l1-transfected HFKs exhibited lower mRNA levels of involucrin, loricrin and caspase-14 when compared with non-Tcf7l1-transfected HFKs, although calcium induced expression of these markers (Figure 5e).

### LCN2 inhibition abrogates effect of Tcf7l1 on keratinocyte differentiation

**Figure 6:**
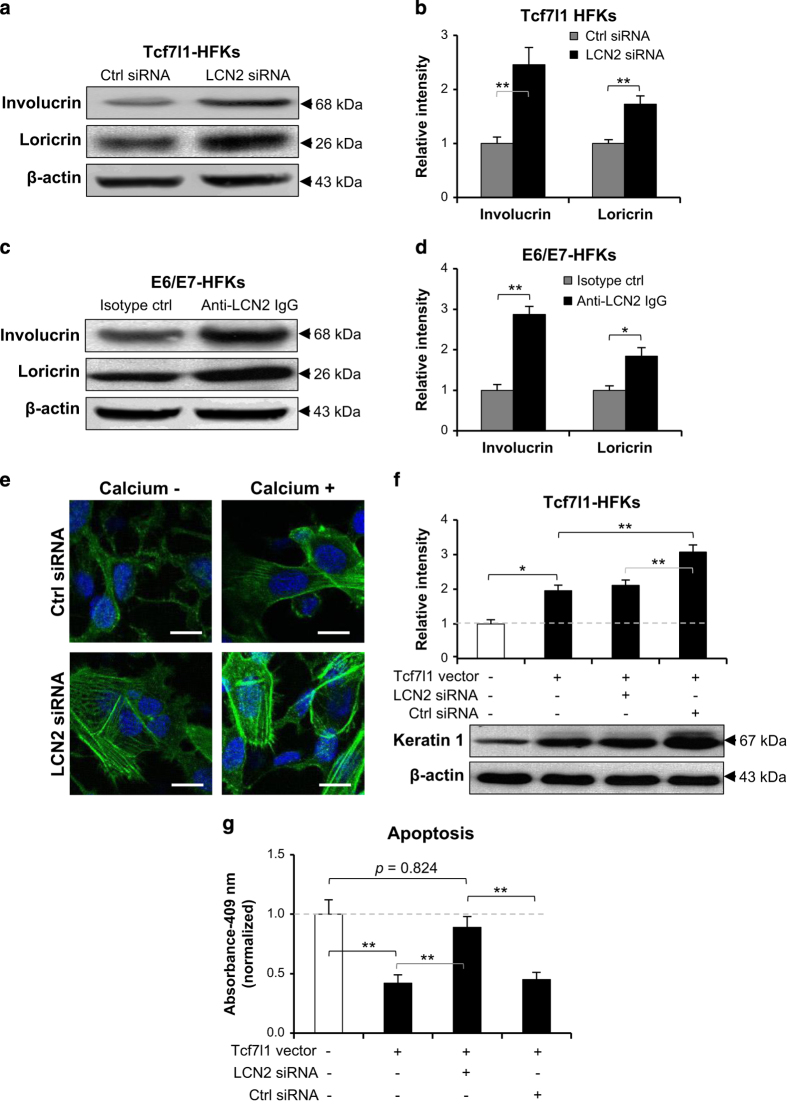
The effect of LCN2 inhibition on HFKs. (**a** and **b**) By western blot, involucrin and loricrin expression was determined in Tcf7l1-transfected HFKs that were then treated with LCN2 or control siRNA. (**c** and **d**) Similarly, involucrin and loricrin were assessed in E6/E7-transfected HFKs that were treated with neutralizing anti-LCN2 antibody or isotype control (2 *μ*g/ml, 2 days). (**e** and **f**) By immunofluorescence (**e**) and western blot (**f**), keratin 1 was detected in HFKs that were transfected with Tcf7l1 vector and then LCN2 or control siRNA. (**g**) HFKs were pre-transfected with Tcf7l1 vector, and then transfected with LCN2 siRNA. Cell apoptosis was quantitated by the CaspaTag caspase 3, 7 method. Data show mean±S.E.M. of triplicate cultures (*n*=3). Statistical differences were tested with one-way ANOVA followed by a two-tailed *t*-test. Representative images are shown. Scale bar=10 *μ*m. *P<0.05; **P<0.01.

Since both Tcf7l1- and E6/E7-vector-transfected HFKs overexpressed Tcf7l1 (Figure 2), LCN2 signals were inhibited in these cells by LCN2 siRNA or neutralizing anti-LCN2 antibody. By western blot, LCN2 inhibition enhanced both involucrin and loricrin expression in Tcf7l1-overexpressing HFKs (Figures 6a–d), which was primarily suppressed in these cells when compared with control HFKs (Supplementary Figure S2 and Figure 5e). Furthermore, the treatment with LCN2 siRNA prompted keratin 1 expression in the Tcf7l1 vector-transfected HFKs (Figures 6e and f). Such effect of LCN2 siRNA transfection on these differentiation markers was also reflected by qRT-PCR analysis (Supplementary Figure S4). Meanwhile, LCN2 siRNA had no effect on the expression of Tcf7l1 mRNA in these HFKs (data not shown). Consistently, LCN2 siRNA recovered both cell apoptosis (Figure 6g) and keratin 1 expression (Supplementary Figure S4) in HFKs that were suppressed by Tcf7l1 overexpression.

### MMP-2 mediates the enhancement effect of Tcf7l1 on LCN2 expression

**Figure 7:**
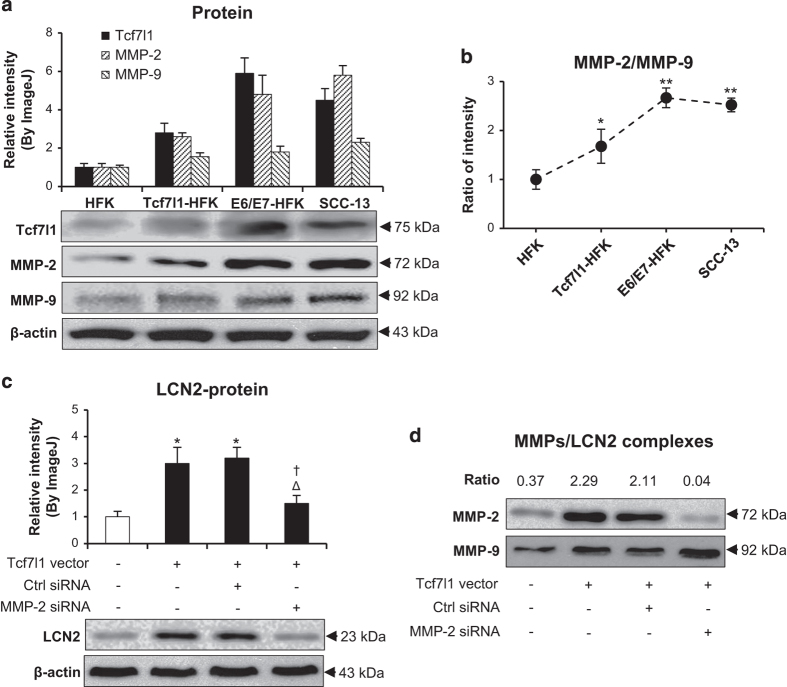
The effect of MMP-2 on LCN2 expression in cells. (**a**) By western blot, the expression of Tcf7l1, MMP-2 and MMP-9 was determined in SCC-13 cells and Tcf7l1- or E6/E7-transfected HFKs. (**b**) The ratios of MMP-2 to MMP-9 were calculated by using the relative intensity values from western blot. *P<0.05, **P<0.01, compared with the control HFK group. (**c**) By western blot, the LCN2 protein was detected in the HFKs that were transfected with Tcf7l1-expressing lentiviral vector or MMP-2/control siRNA. *P<0.05, compared with the nontreated group; ^Δ^*p*<0.05, compared with Tcf7l1 transfection alone group; ^†^*P*<0.05, compared with control siRNA group. (**d**) The MMP-2 and MMP-9 proteins were blotted in cell lysates that were precipitated by anti-LCN2 IgG-conjugated protein IgG agarose. The ratios mean MMP-2 to MMP-9 values. Data show mean±S.E.M. of triplicate cultures (*n*=3). Statistical differences were tested with one-way ANOVA followed by a two-tailed *t*-test.

The proteins of MMP-2 and MMP-9 were determined by western blot in SCC-13 cells and Tcf7l1- or E6/E7-transfected HFKs. The results showed that both MMP-2 and MMP-9 levels increased with Tcf7l1 expression (Figure 7a). However, the MMP-2 level was more affected by Tcf7l1, leading to an increase in the MMP-2/MMP-9 ratio in Tcf7l1-overexpressing cells (Figure 7b). In addition, the transfection of MMP-2 but not control siRNA significantly reduced LCN2 production in Tcf7l1-overexpressing HFKs (Figure 7c). Consistently, the mRNA levels of Tcf7l1, MMP-2, MMP-9 and LCN2 showed a similarity to the proteins in these cells (Supplementary Figure S5).

The MMPs/LCN2 complexes in cell lysates were detected by immunoprecipitation and then determined by western blot. Interestingly, the anti-LCN2 IgG-precipitated samples revealed more MMP-2 and higher MMP-2/MMP-9 ratio in the Tcf7l1-overexpressing HFKs when compared with control HFKs (Figure 7d).

## Discussion

In the present study, we found that Tcf7l1 and LCN2 are overexpressed in the keratinocytes and skin diseases that are characterized by dysregulated differentiation. Moreover, LCN2 expression increases with the Tcf7l1 level in HFKs undergoing differentiation, and decreases significantly upon Tcf7l1 depletion. Furthermore, Tcf7l1 overexpression induces suppression of both cell apoptosis and differentiation marker expression, which can be partially abrogated by LCN2 inhibition. Finally, Tcf7l1 overexpression induces more MMP-2 than MMP-9 in HFKs, and MMP-2 inhibition represses the LCN2 level. Therefore, these results convincingly demonstrated that Tcf7l1 plays a pivotal role in the dysregulation of keratinocyte differentiation through upregulating the LCN2 pathway.

Our results are consistent with previous reports. Lee *et al.*^25^ found stronger staining of LCN2 in lichen planus, pityriasis rubura pilaris, keratoacanthoma and SCC than in pityriasis rosea and BCC. Mallbris *et al*. demonstrated a higher LCN level in psoriasis than in healthy controls.^26^ Nguyen *et al*.^4^ even found that Tcf7l1 induces LCN2 gene expression in epidermis isolated from mice. In the present study, we further supported these findings by not only providing quantitative data but also showing more LCN2 expression in verrucous epidermal nevus and condyloma acuminatum, which both harbor dysregulation of keratinocyte differentiation (parakeratosis) as other disorders above. In addition, we verified a positive correlation between Tcf7l1 and LCN2 expression in these tissues. Impressively, Tcf7l1 increases in terms of Broders’ classification grade of SCC, further indicating involvement of Tcf7l1 in cell differentiation. The overexpression of Tcf7l1 and LCN2 in melanocytes of pigmented nevus suggests an additional function of the Tcf7l1–LCN2 axis, which should be elucidated in other studies.

In this study, HFKs were induced for differentiation by calcium stimuli. It had been found that LCN2 is calcium inducible in keratinocytes and increases with stimulation time.^25^ We saw elevated Tcf7l1 and LCN2 expression in HFKs under calcium stimulation, which seems to bring doubt on the effect of Tcf7l1 on keratinocyte differentiation since involucrin and loricrin, two terminal differentiation markers,^27^ are also enhanced upon calcium stimulation.^28^ We speculated that HFKs undergoing differentiation synthesize involucrin and loricrin naturally, which, however, induce Tcf7l1 and LCN2 expression in a feedback manner. In other words, Tcf7l1 and LCN2 control the overfunction of involucrin and loricrin in keratinocytes during differentiation. Such speculation was verified by the facts that in HFKs under calcium stimulation, Tcf7l1 overexpression reduces the levels of involucrin, loricrin and caspase-14 while Tcf7l1 depletion promotes involucrin and loricrin production.

As specific markers for cell differentiation, involucrin, loricrin and caspase-14 increase in keratinocytes during differentiation^27,29^ but decrease during dysregulation of keratinocyte differentiation.^30,31^ Our results showed an enhancement effect of Tcf7l1 depletion on these markers as well as a reverse effect of Tcf7l1 overexpression on them, unveiling negative regulation of Tcf7l1 in keratinocyte differentiation. Another important feature of differentiation dysregulation in keratinocytes is the decrease in apoptosis while proliferation may change or not.^32,33^ We found that Tcf7l1 overexpression inhibits apoptosis of HFKs but shows no effect on cell proliferation, also reflecting the role of Tcf7l1 in dysregulating differentiation. Additionally, the blockade of LCN2 downregulates the expression of keratin 1 in calcium-stimulated HFKs, providing more evidence in support of our speculation.

In both skin tissues and keratinocytes that have different status of differentiation, LCN2 expression fluctuates with the Tcf7l1 level, revealing a positive correlation between the two proteins. Importantly, the inhibition of LCN2 through the siRNA approach or anti-LCN2 antibody abrogates the effect of Tcf7l1 on keratinocyte differentiation, further confirming a LCN2 mediation in Tcf7l1-dysregulated keratinocyte differentiation. Considering many studies that demonstrated the critical role of LCN2 in suppressing cell differentiation,^12,14,18^ we deduced that the Tcf7l1–LCN2 axis is effective in dysregulating keratinocytes.

An interesting finding in this study was that Tcf7l1 induces high level of MMP-2 and enhances the MMP-2/MMP-9 ratio in HFKs. It has been known that both MMP-2 and MMP-9 are involved in cell differentiation.^34–36^ However, MMP-9 promotes terminal differentiation of keratinocytes while MMP-2 counters the effect of MMP-9 and prevents keratinocytes from such differentiation.^37^ Previous studies demonstrated that the complexes of LCN2 plus MMP-2 or MMP-9 form in tissues,^20,38,39^ and stabilize the partners by protecting autodegradation.^40,41^ Additionally, we found that Tcf7l1 enhances MMP-2 bound by LCN2, and MMP-2 inhibition reduces effect of Tcf7l1 on the expression of LCN2 and some markers for keratinocyte differentiation. Therefore, the prevailing MMP-2 expression in Tcf7l1-overexpressing HFKs may favor LCN2 upregulation in a feedback manner, and subsequently leads to the dysregulation of keratinocyte differentiation. Currently, the precise mechanism underlying the MMP-2 mediation of LCN2 upregulation is unclear, and deserves further study.

In conclusion, this study demonstrated the overexpression of Tcf7l1 and LCN2 in keratinocytes with dysregulated differentiation. Tcf7l1 enhances the expression of MMP-2 in keratinocytes, which further upregulates the level of LCN that correlates closely with differentiation markers. Therefore, LCN2 mediates the Tcf7l1 dysregulation of keratinocyte differentiation. Targeting the Tcf7l1–LCN2 axis may be one of the approaches in managing skin diseases with dysregulated keratinocyte differentiation.

## Materials and Methods

### Tissue samples

The tissue samples were collected from healthy donors or patients with seborrheic keratosis, psoriasis vulgaris, verrucous epidermal nevus, pigmented nevus, Bowenoid papulosis, BCC, condyloma acuminatum and SCC, who had been diagnosed pathologically but received no therapy within the past 4 weeks (Supplementary Table S1). These samples were processed for paraffin sections, whereas some fresh tissues were surgically dissected of subcutaneous fat before freezing at −80 °C.^42^ Based on hematoxylin and eosin staining, SCC samples were graded according to Broders’ classification: grade I, tumors in which more than 75% of cells are differentiated; grade II, tumors with 50–75% differentiation; grade III, tumors with 25–50% differentiation; and grade IV, tumors with less than 25% differentiation.^43^ This study was conducted under the supervision of the research ethics committees of the hospitals. Written informed patient consent was obtained before harvesting the tissue samples.

### Immunohistochemistry

As described previously,^44^ paraffin sections were routinely dewaxed, and the antigens were retrieved by using a microwave method. After permeabilization with 0.1% Triton-100/PBS, sections were blocked with dual endogenous enzymes (DAKO, Glostrup, Denmark) and then incubated with rabbit anti-Tcf7l1 or LCN2 IgG (2 *μ*g/ml; Santa Cruz, Dallas, TX, USA). Thereafter, the horseradish peroxidase (HRP)-labeled polymer-conjugated goat anti-rabbit IgG (DAKO) was added to sections before the color (brown) development with the 3,3'-diaminobenzidine-chromogen substrate. Finally, the sections were counterstained with Mayer’s hematoxylin.

### Cell culture and *in vitro* transfection

SCC-13 cells were grown in Dulbecco's modified eagle media with 10% FBS.^45^ HFKs were isolated from neonatal foreskin,^46^ and cultured in the same media for SCC-13 cells. The retroviral infection of HPV16 E6/E7 genes was performed on HFKs.^46^ By following a reported method,^47^ human Tcf7l1 cDNA was subcloned into a pDC315 vector (gifted by Dr. Cheng Pan, Wuhan University) and then transfected into HFKs. Using gene-specific PCR primers (Supplementary Table S2), Tcf7l1 cDNA was incorporated with sequences of *Eco*RI and *Nhe*I, and then ligated into the corresponding restriction enzyme sites in vector. Tcf7l1 overexpression in transfected cells were verified by both qRT-PCR and western blot. In some experiments, calcium chloride (1.2 mM), neutralizing anti-LCN2 antibody or isotype control (R&D Systems, Minneapolis, MN, USA) were added to cultured media.

siRNA transfection was carried out as reported previously.^48^ The predesigned Tcf7l1, LCN2, MMP-2 or control siRNA (Life Technologies, Carlsbad, CA, USA; Supplementary Table S2) was dissolved in a lipofectamine 2000 reagent at a ratio of 10 pmol per *μ*l. The final concentrations of siRNAs in media were 15 pmol/ml. qRT-PCR was used for verifying such transfection. Cells were used for assays 3 days after transfection.

### Immunofluorescence and flow cytometry

Immunofluorescent detection was performed as described previously.^49^ Cells grown on cover glass were fixed with 4% paraformaldehyde solution. After blocking, cells were incubated with rabbit anti-Tcf7l1 (fluorescein isothiocyanate-conjugated; Jieqing Co., Wuhan, China) or anti-LCN2 (Alexa Fluor 555-conjugated, Bioss Inc., Woburn, MA, USA) IgG. For confocal analysis, rabbit anti-involucrin (Alexa Fluor 555-conjugated; Bioss), anti-loricrin (Cy5-conjugated) (Biorbyt LLC, San Francisco, CA, USA) or anti-cytokeratin 1 (Alexa Fluor 488-conjugated) (Abcam, Cambridge, MA, USA) antibodies were used by following the standard method.^50^ In flow cytometry, these antibodies were used also but detected in a LSRII instrument (BD Biosciences, San Jose, CA, USA). Data were analyzed using FlowJo7.6.1 software (Tree Star, Ashland, OR, USA).

### Cell apoptosis and proliferation

The apoptosis and proliferation of cells were quantitatively analyzed by using a CaspaTag caspase 3,7 fluorescein assay kit (EMD Millipore, Billerica, MA, USA) and a CellTiter 96 solution (Promega Co., Madison, WI, USA), respectively.^42^ Besides, cell apoptosis and proliferation were visualized by terminal deoxynucleotidyl transferase-mediated dUTP nick end labeling (Jieqing Co.) and Alexa Fluor 647-labeled Ki-67 staining (Abcam), respectively.

### qRT-PCR

qRT-PCR was performed as described previously.^51^ By using PureLink RNA kit (Invitrogen, Grand Island, NY, USA), total RNA was extracted from fresh tissues or cell cultures. Reverse transcription was carried out with a commercial cDNA kit (Applied Biosystems, Carlsbad, CA, USA). Amplification was carried out using the 7900HT Fast PCR System (Applied Biosystems). SYBR Green Master Mixes (Invitrogen) was used as the fluorescent dye. The sequences of primers (Invitrogen) are listed in Supplementary Table S2.

### Western blotting and immunoprecipitation

Protein lysates were extracted using a RIPA lysis buffer supplemented with a protease inhibitor cocktail (Thermo Scientific, Shanghai, China). Western blot was performed according to a previous protocol.^52^ Proteins were separated by electrophoresis, and then transferred onto polyvinylidene difluoride membranes. The rabbit primary antibodies (all from Santa Cruz) were used, followed by incubation with HRP-labeled goat anti-rabbit IgG (Southern Biotech, Birmingham, AL, USA). Signals were developed with an ECL kit (Thermo Scientific). All normalized intensities were quantified by Image J1.61 u.

For immunoprecipitation, cell lysates were incubated with protein G agarose and anti-LCN2 IgG as described previously.^20^ The bound complexes were washed from agarose beads, and then analyzed by western blot at a reducing and denatured condition.

### ELISA

LCN2 in the culture supernatants was detected by using a Quantikine sandwich ELISA kit (R&D Systems), according to the manufacturer’s instruction.

### Statistical analysis

Data were presented as the mean±standard error of the mean (S.E.M.). Statistical analysis was performed using the STATA 10.0 software (StataCorp., College Station, TX, USA). ANOVA was used in the comparison of more than two groups. Data were analyzed by one-way ANOVA followed by a two-tailed *t*-test. Linear regression was done for the correlation between the mRNA expression values of Tcf7l1 and LCN2. The differences were considered significant at *P*<0.05.

## Abbreviations

BCC: basal cell carcinoma
ELISA: enzyme-linked immunosorbent assay
HFK: human foreskin keratinocyte
HPV: human papilloma virus
LCN2: lipocalin 2
MMP: matrix metalloproteinase
qRT-PCR: quantitative real-time polymerase chain reaction
SCC: squamous cell carcinoma
Tcf7l1: transcription factor 7-like 1.
