# Supplementary figures and images for: Transcription factor 7-like 1 dysregulates keratinocyte differentiation through upregulating lipocalin 2
Source: Cell Death Discov. 2016 Apr 25;2:16028–. doi: 10.1038/cddiscovery.2016.28 (PMC4979464; doi:10.1038/cddiscovery.2016.28)

Figure S1

**a**

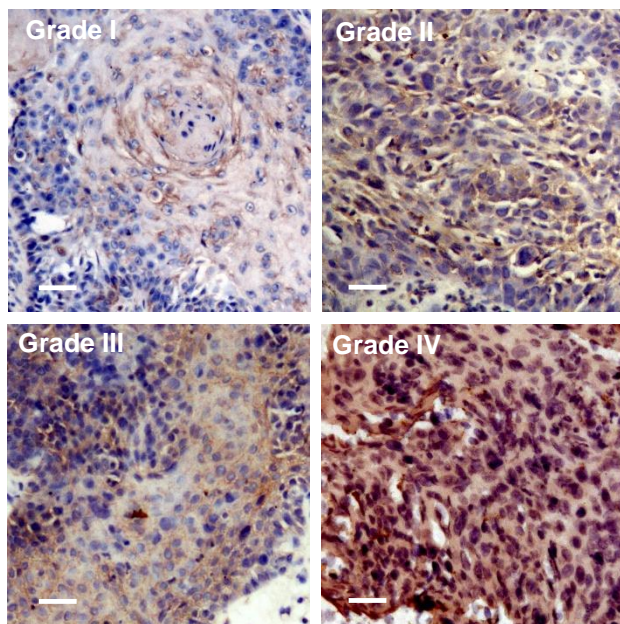

**b**

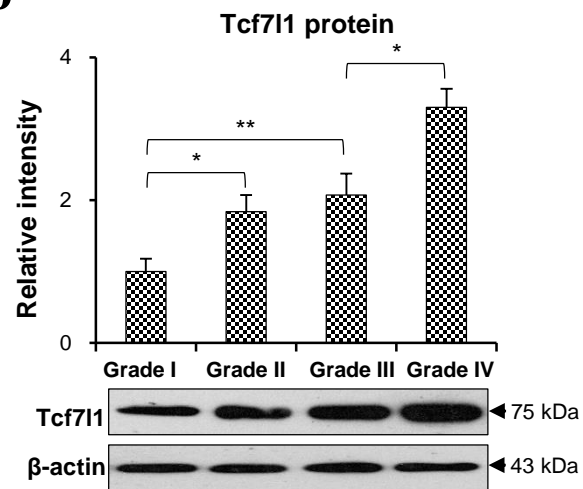

**c**

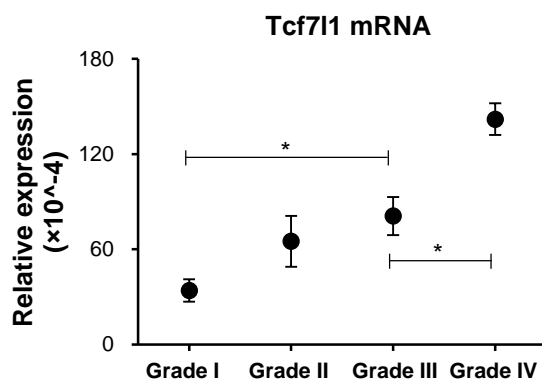

Figure S2

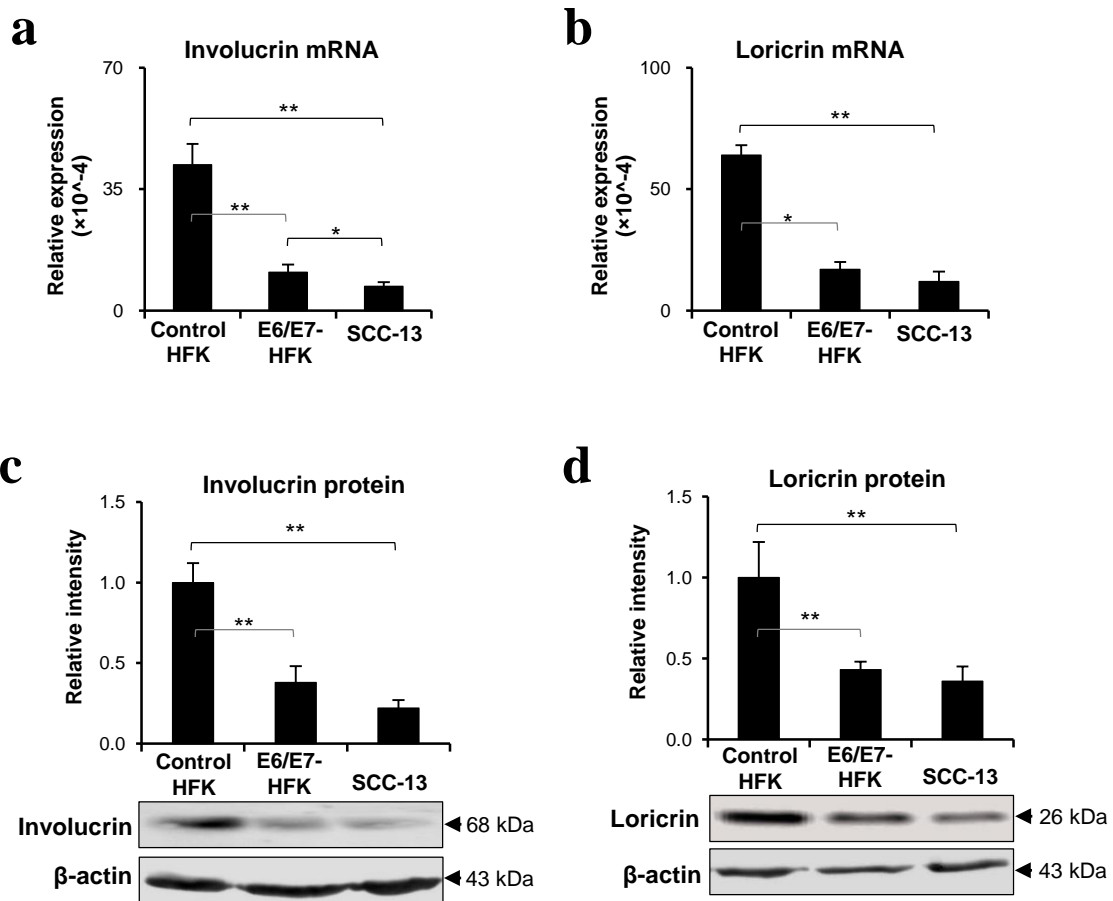

Figure S3

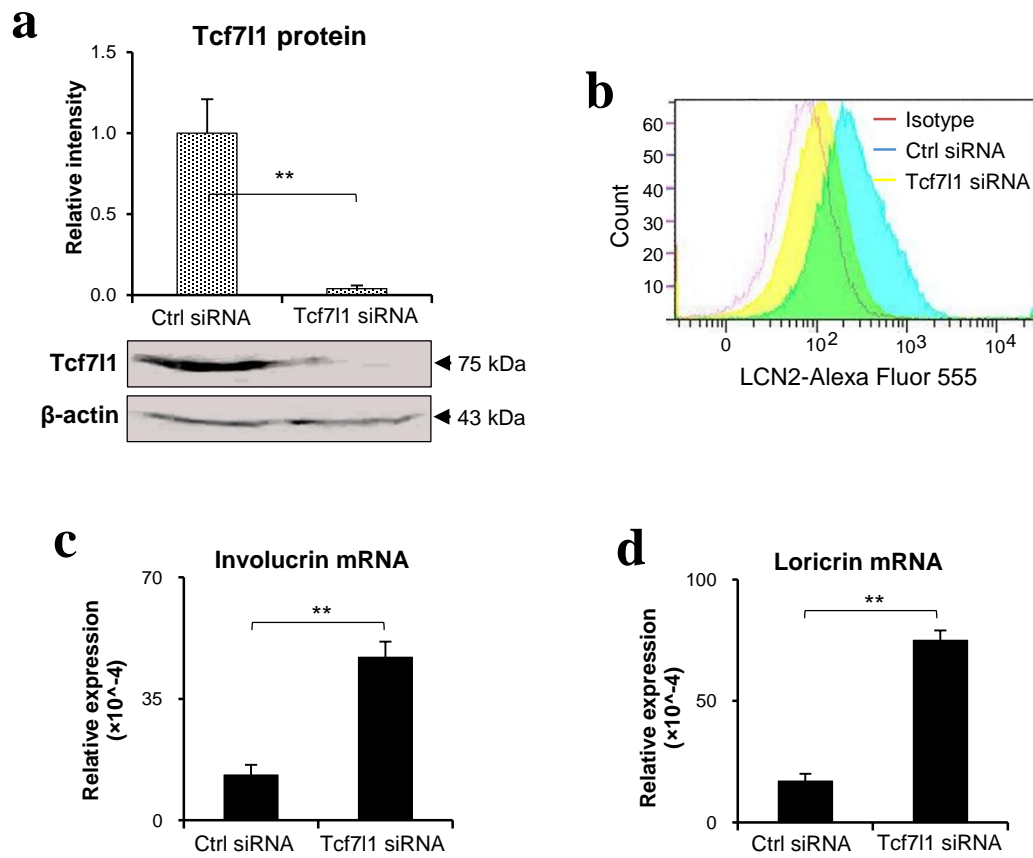

Figure S4

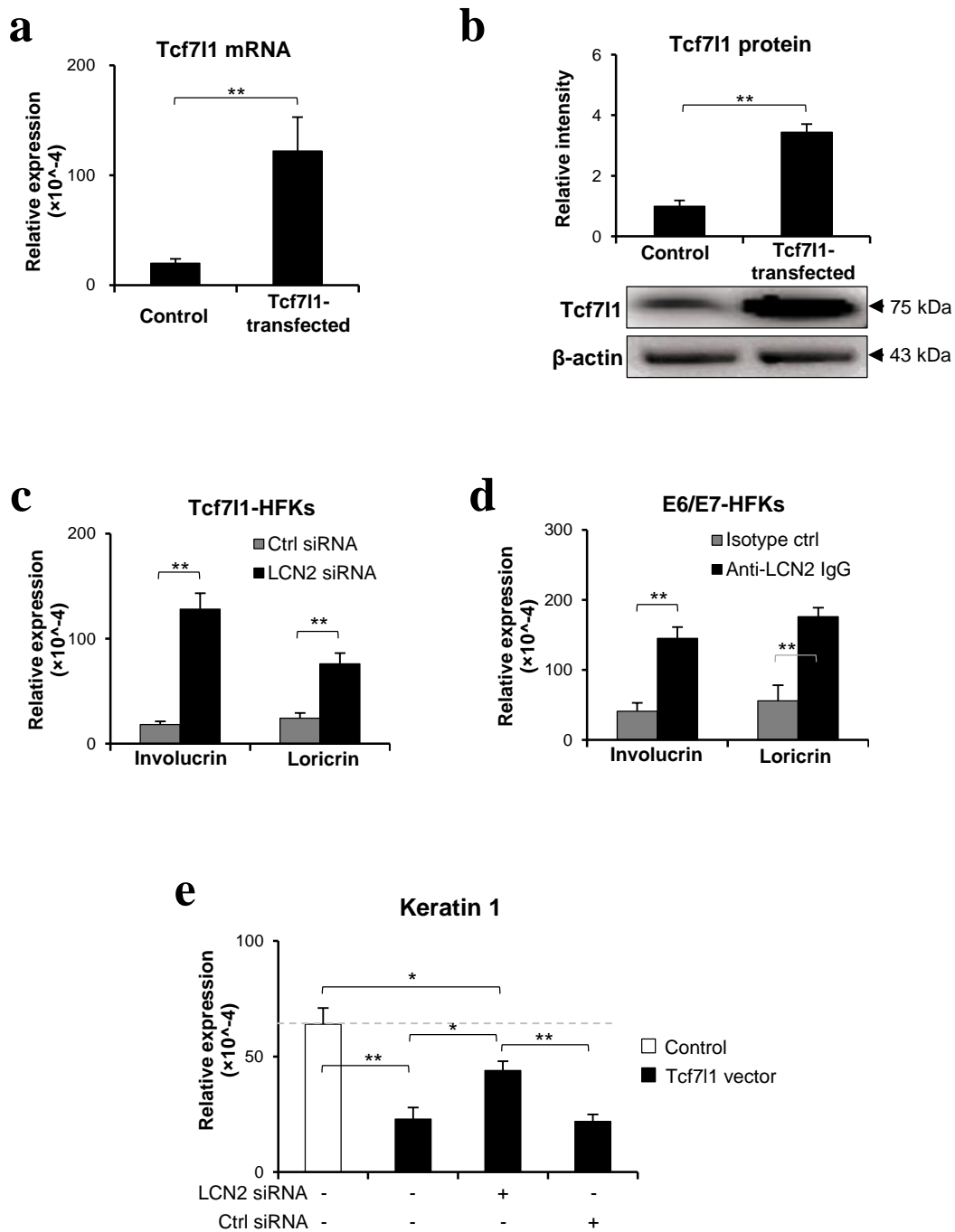

Figure S5

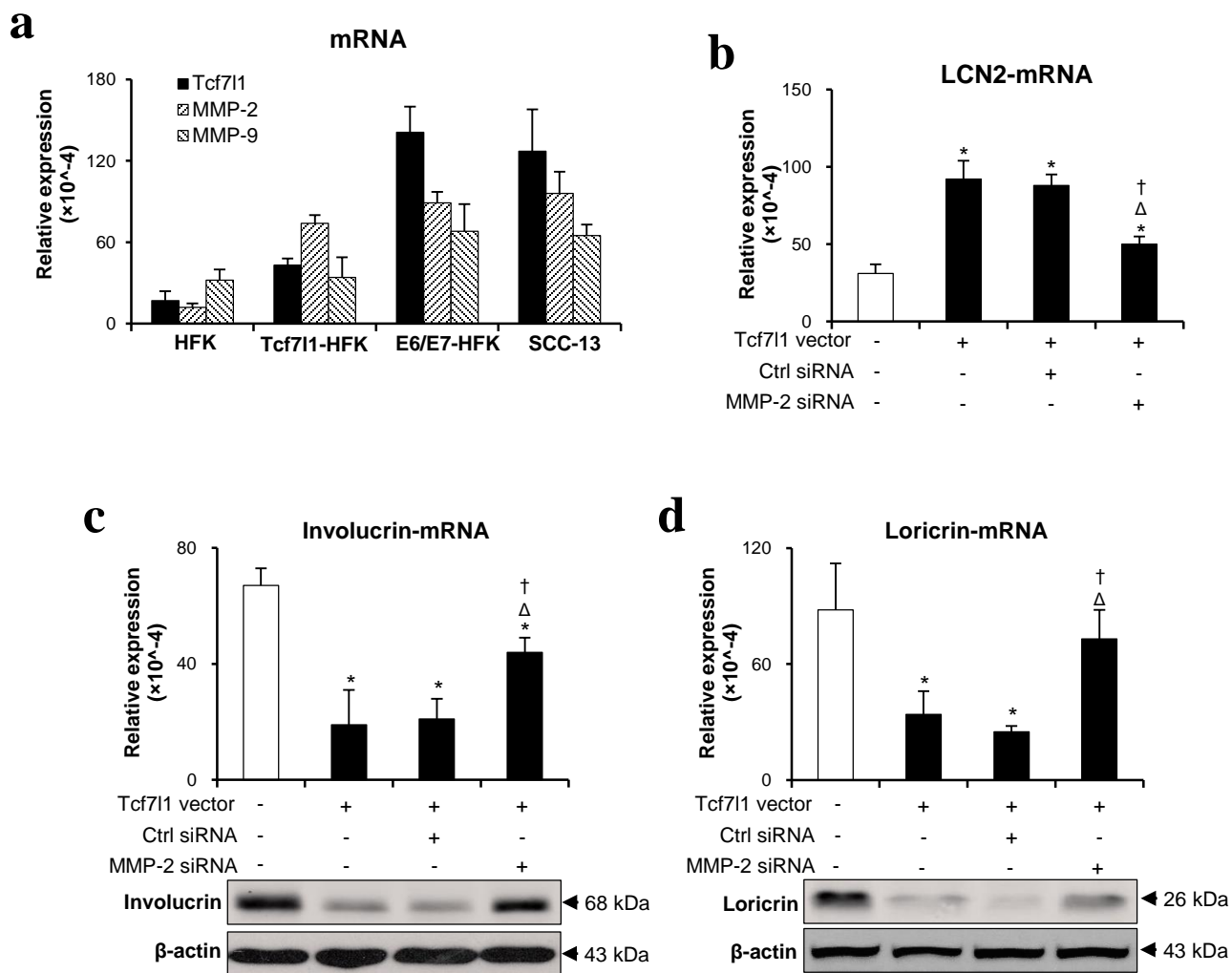

Supplement: Supplementary Figures [file cddiscovery201628-s1.pdf]
