# Supplementary material for: Transcription factor 7-like 1 dysregulates keratinocyte differentiation through upregulating lipocalin 2
Source: Cell Death Discov. 2016 Apr 25;2:16028–. doi: 10.1038/cddiscovery.2016.28 (PMC4979464; doi:10.1038/cddiscovery.2016.28)
Supplement: Supplementary Information [file cddiscovery201628-s2.doc]

**Supplementary Figure 1** Tcf7l1 expression in SCC of different differentiation. (**a**) By immunohistochemistry, Tcf7l1 was detected in paraffin sections of SCC samples with differentiation grades from I to IV. (**b**) By Western blot, Tcf7l1 was determined in protein extractions from fresh SCC tissues. (**c**) The mRNA expression of Tcf7l1 was analyzed in fresh SCC tissues. The numbers of tissue samples were 3 (grade I), 4 (grade II), 3 (grade III), and 3 (grade IV), respectively. Data show mean ± S.E.M. of triplicate cultures (n = 3). Statistical differences were tested with one-way ANOVA followed by a two-tailed *t*-test. Representative images are shown. Scale bars = 25 μm. **p* < 0.05; ***p* < 0.01.

**Supplementary Figure 2** The involucrin and loricrin expression in different keratinocyte-originated cells. The total RNA and proteins were extracted from control (transfected with blank vector) human foreskin keratinocytes (HFKs), E6/E7-transfected HFKs, and SCC-13 cells. (**a** and **b**) The mRNA levels of involucrin (**a**) and loricrin (**b**) were determined by quantitative real-time polymerase chain reaction. (**c** and **d**) The protein expression of involucrin (**c**) and loricrin (**d**) was determined by Western blot. Intensities of Western blot bands were quantitated by ImageJ. Data show mean ± S.E.M. of triplicate cultures (n = 3). Statistical differences were tested with one-way ANOVA followed by a two-tailed *t*-test. Representative images are shown. **p* < 0.05; ***p* < 0.01.

**Supplementary Figure 3** The effect of Tcf7l1 depletion on LCN2, involucrin, and loricrin expression in HFKs. HFKs were transfected with Tcf7l1 or control siRNA before calcium stimulation (1.2 mM, 7 days). (**a**) Tcf7l1 inhibition was verified by both Western blot. (**b**) LCN2 expression was analyzed by flow cytometry upon siRNA transfection. (**c** and **d**) The mRNA levels of involucrin (**c**) and loricrin (**d**) were determined in these cells. Intensities of Western blot bands were quantitated by ImageJ. Data show mean ± S.E.M. of triplicate cultures (n = 3). Statistical differences were tested with a two-tailed *t*-test. Representative images are shown. ***p* < 0.01.

**Supplementary Figure 4** The effect of LCN2 inhibition on differentiation markers. (**a** and **b**) The Tcf7l1 cDNA-inserted pDC315 vector was transfected into HFKs, followed by the determination of Tcf7l1 mRNA (**a**) and protein (**b**) expression. (**c**) By qRT-PCR, the mRNA levels of involucrin and loricrin were determined in Tcf7l1-transfected HFKs that were then transfected with LCN2 or control siRNA. (**d**) The mRNA levels of involucrin and loricrin were determined in E6/E7-positive HFKs that were treated with neutralizing anti-LCN2 antibody or isotype control (2 µg/ml, 2 days). (**e**) The mRNA expression of keratin 1 was determined in the HFKs that were transfected with Tcf7l1 vector and then LCN2 or control siRNA. Data show mean ± S.E.M. of triplicate cultures (n = 3). Statistical differences were tested with one-way ANOVA followed by a two-tailed *t*-test. Representative images are shown. **p* < 0.05; ***p* < 0.01.

**Supplementary Figure 5** The effect of MMP-2 inhibition on Tcf7l1-regulated genes. (**a**) By qRT-PCR, the mRNA levels of Tcf7l1, MMP-2, and MMP-9 were determined in HFKs and SCC-13 cells. (**b**) The mRNA expression of LCN2 was determined in the HFKs that were transfected with Tcf7l1 vector and then MMP-2 or control siRNA. (**c** and **d**) Similarly, the mRNA levels of involucrin (**c**) and loricrin (**d**) were determined in these HFKs. Data show mean ± S.E.M. of triplicate cultures (n = 3). Statistical differences were tested with one-way ANOVA followed by a two-tailed *t*-test. **p* < 0.05, compared to the nontreated group; Δ*p* < 0.05, compared to Tcf7l1 transfection alone group; †*p* < 0.05, compared to control siRNA group.

**Supplementary Table 1. Demographic characteristics of the patients with skin disorders**

| Skin disease | Number  (F/M) | Age  (years, mean ± SEM) | Duration  (months, mean ± SEM) |
| --- | --- | --- | --- |
| SCC | 8/5 | 63.20 ± 5.84 | 3.11 ± 1.37 |
| BCC | 1/3 | 70.44 ± 8.59 | 9.23 ± 3.20 |
| Seborrheic keratosis | 3/1 | 63.75 ± 9.52 | 33.17 ± 7.24 |
| Bowenoid papulosis | 3/0 | 52.83 ± 8.30 | 17.26 ± 7.38 |
| Psoriasis vulgaris | 2/3 | 46.36 ± 6.91 | 23.12 ± 8.57 |
| verrucous epidermal nevus | 1/2 | 52.00 ± 11.25 | 62.22 ± 15.46 |
| Pigmented nevus | 3/1 | 35.93 ± 7.77 | 58.75 ± 21.16 |
| condyloma acuminatum | 2/2 | 28.42 ± 5.70 | 3.54 ± 1.39 |
| Normal skin | 2/2 | 36.85 ± 10.19 | None |

Note: F, female; M, male; BCC, basal cell carcinoma; SCC, squamous cell carcinoma; SEM, standard error of the mean.

**Supplementary Table** 2. Sequences of oligonucleotides

| Oligonucleotide | Sequence | Size of product |
| --- | --- | --- |
| Caspase-14 primers | Forward: 5′-ATTCCCTGTTGTCACCTTGC-3′  Reverse: 5′-ACCTGGAGGTAACCTTGGCT-3′ | 304 bp |
| GAPDH primers | Forward: 5′-GGTGTGAACCATGAGAAGTATGA-3′  Reverse: 5′-GAGTCCTTCCACGATACCAAAG-3′ | 124 bp |
| Involucrin primers | Forward: 5′-AAAGCAGAAAACCCAGAGCA-3′  Reverse: 5′-CTCTAGGTGCTTCAGGTGCC-3′ | 224 bp |
| LCN2 primers | Forward: 5′-CAAGGAGCTGACTTCGGAAC-3′  Reverse: 5′-GCCTGAGGGCACATGTTTAT-3′ | 286 bp |
| Loricrin primers | Forward: 5′-CATGATGCTACCCGAGGTTT-3′  Reverse: 5′-ACTGGGGTTGGGAGGTAGTT-3′ | 152 bp |
| MMP-2 primers | Forward: 5′-TCCCATTTTGATGACGATGA-3′  Reverse: 5′-CCGTACTTGCCATCCTTCTC-3′ | 203 bp |
| MMP-9 primers | Forward: 5′-GAGTTCCCGGAGTGAGTTGA-3′  Reverse: 5′-ACTCCTCCCTTTCCTCCAGA-3′ | 193 bp |
| Tcf7l1 primers | Forward: 5′-TTCGTCCTGTAGGTGCTGTG-3′  Reverse: 5′-ACCCACGGTATTGAGCAGAC-3′ | 197 bp |
| Control siRNA | Life Technologies, Cat. #AM4611 (Negative control) | / |
| MMP-2 siRNA | Life Technologies, Cat. #113125 | / |
| LCN2 siRNA | Life Technologies, Cat. #121012 | / |
| Tcf7l1 siRNA | Life Technologies, Cat. #116427 | / |
| Tcf7l1 ligation | Forward: 5′-ATATAGAATTCGGCGGCCCCGGCCCGCGGCCCC-3′ | 1839 bp |
|  | Reverse: 5′-ATATAGCTAGCACAGCCTGCAGGGGTCGGGGGG-3′ |  |

Note: GAPDH, glyceraldhyde-3-phosphate dehydrogenase; LCN2, lipocalin 2; MMP, matrix metalloproteinase; Tcf7l1, transcription factor 7-like 1.
